# Incidence et déterminants du rebond de la charge virale chez les personnes sous dispensation multi mois d’antirétroviraux à l’Hôpital Régional Annexe de Dschang de 2018-2023

**DOI:** 10.11604/pamj.2024.49.74.45348

**Published:** 2024-11-12

**Authors:** Elvira Francheska Kengni, Djerry Dunhill Nzapze, Cavin Epie Bekolo, Charles Kouanfack

**Affiliations:** 1 Faculty of Medicine and Pharmaceutical Sciences, University of Dschang, Dschang, Cameroon,; 2 Global Research Agency, Dschang, Cameroon

**Keywords:** VIH, rebond viral, dispensation multi-mois, facteurs associés, HIV, viral rebound, multi-month dispensing, associated fact

## Abstract

**Introduction:**

au Cameroun, la dispensation multi-mois (DMM) des antirétroviraux (ARV) a été introduite pour améliorer l'adhésion thérapeutique des personnes vivant avec le VIH (PVVIH). Cependant, cette approche présente des limites susceptibles de favoriser un rebond de la charge virale. Cette étude vise à évaluer l'incidence et les facteurs associés au rebond viral chez les PVVIH sous DMM à l'Hôpital Régional Annexe de Dschang entre 2018 et 2023.

**Méthodes:**

il s'agit d'une étude de cohorte rétrospective couvrant la période de janvier 2018 à décembre 2023, comparant l'incidence du rebond viral (augmentation de plus de 100 copies/ml après suppression) et ses déterminants chez les patients sous DMM et ceux sous dispensation mensuelle (DM). Les données ont été collectées à partir des dossiers médicaux au moyen d´une grille standardisée. Des statistiques descriptives ont été suivies du calcul des Odds Ratios pour identifier les facteurs associés au rebond viral, avec un seuil de signification de p<0,05.

**Résultats:**

un total de 519 patients (438 sous DMM et 81 sous DM) ont été inclus. L'incidence du rebond viral était de 18,8 % chez les patients sous DMM, contre 37,8% chez ceux sous DM. Le ratio homme/femme était de 0,53, avec une majorité de patients âgés de 30 à 45 ans. La couverture de la DMM est passée de 76% en 2018 à 51,02 % en 2023. Les facteurs significativement associés au rebond viral étaient le type de dispensation (p=0,001), l'interruption du traitement (p=0,001), l'âge (30-45 ans; p=0,001) et le tabagisme (p=0,008).

**Conclusion:**

l'incidence du rebond viral est plus élevée chez les patients sous DM. Il est essentiel d'améliorer la gestion des PVVIH et de promouvoir de meilleures habitudes de vie pour prévenir l'échec thérapeutique à long terme.

## Abstract (en)

**Introduction:**

in Cameroon, multi-month dispensing (MMD) of antiretrovirals (ARVs) was introduced to improve treatment adherence among people living with HIV (PLHIV). However, this strategy has limitations that may lead to viral load rebound. The purpose of this study is to assess the incidence and factors associated with viral rebound in PLHIV receiving MMD at the Dschang Regional Annex Hospital between 2018 and 2023.

**Methods:**

we conducted a retrospective cohort study comparing the incidence of viral rebound (increase of over 100 copies/mL after suppression) and its determinants in patients on MMD versus those on monthly dispensing (MD) from January 2018 to December 2023. Data were collected from patients´ medical records using a standardized data collection sheet. Descriptive statistics were followed by bivariate and multivariate analyses to identify factors associated with viral rebound, with a significance threshold of p<0.05.

**Results:**

a total of 519 patients (438 on MMD and 81 on MD) were included. The incidence of viral rebound was 18.8% among patients on MMD compared to 37.8% among those on MD. The male-to-female ratio was 0.53, with the majority of patients aged 30 to 45 years. MMD coverage decreased from 76% in 2018 to 51.02% in 2023. Factors significantly associated with viral rebound included dispensing type (p=0.001), treatment interruption (p=0.001), age group 30-45 years (p=0.001) and tobacco use (p=0.008).

**Conclusion:**

the incidence of viral rebound is higher among patients on MD. Improving the management of PLHIV and promoting healthier lifestyle choices are essential to prevent long-term treatment failure.

## Introduction

Le virus de l´immunodéficience humaine (VIH) constitue une menace persistante pour la santé mondiale en raison de son impact dévastateur sur le système immunitaire, entraînant souvent le syndrome d´immunodéficience acquise (SIDA) dans sa phase avancée [1]. Malgré les progrès significatifs dans la prise en charge du VIH/SIDA, cette maladie demeure l'une des principales causes de morbidité et de mortalité dans le monde [2]. Selon les statistiques de l'ONUSIDA, environ 39 millions de personnes dans le monde vivaient avec le VIH, dont 1,3% de personnes nouvellement infectées en 2022 et 630 000 décès enregistrés en 2022 [3]. La situation est particulièrement préoccupante en Afrique, où plus de 25,7 millions de PVVIH représentent la majorité des nouvelles infections mondiales [1]. En 2020, la prévalence du Cameroun était de 2,1%. Ce taux est passé de 3,1% en 2020 à 2,1% en 2023 [4]. Dans le cadre de la lutte contre le VIH/SIDA, les Nations Unies ont établi des objectifs ambitieux visant à assurer que 95% des personnes vivant avec le VIH connaissent leur statut, que 95% de celles-ci aient accès au traitement et que 95% de celles qui sont sous traitement aient une suppression virale indétectable d'ici à 2030 [1]. Bien que des progrès aient été réalisés, les statistiques de 2022 indiquent que seulement 86% des PVVIH connaissaient leur statut, 76% avaient accès au traitement et 71% avaient une suppression de la charge virale (CV < 1000 copies/ml) [3]. Au Cameroun, 5% des PVVIH connaissaient leur statut, 74,7% avaient accès au traitement et 88,1% avaient une suppression de la charge virale [5]. Ces résultats soulignent la nécessité d'approfondir notre compréhension des défis persistants dans la prise en charge du VIH/SIDA [6].

Au cours des deux dernières décennies, l'effort mondial de lutte contre le VIH s´est principalement concentré sur l´élargissement de l´accès au traitement antirétroviral (TARV). Cette stratégie a entraîné une augmentation significative de la suppression de la charge virale (CV >1000 copies/ml), contribuant ainsi à une baisse régulière des décès liés au VIH/SIDA [7]. Le test de la charge virale a été recommandé par l'Organisation mondiale de la Santé (OMS) comme approche de surveillance privilégiée pour diagnostiquer et confirmer l'échec du traitement [8,9]. Malgré ces progrès, il est essentiel de reconnaître qu'un nombre non négligeable de personnes, bien qu'ayant atteint une suppression virale initiale, peuvent connaître un rebond viral [10]. Les taux de rebond viral varient considérablement d'une région à l'autre, avec des incidences rapportées de 21% au Ghana, 40% au Kenya, 28,06% au Royaume-Uni et 19,11 pour 1000 au Cameroun [2,10-12]. Cette disparité met en lumière l'importance de comprendre les facteurs sous-jacents à ce phénomène, qui peuvent varier en fonction des contextes socio-économiques, environnementaux et de soins de santé (le type de dispensation mensuelle et de dispensation multi-mois).

Il existe plusieurs types de dispensation mensuelle qui consiste à s´approvisionner en ARV chaque mois. Elle présente certaines limites: risque de rupture de stock, confidentialité du patient, coûts supplémentaires; raison pour laquelle il a été mis sur pied en 2016 la dispensation multi-mois qui, quant à elle est une approche stratégique consistant à prescrire des ARVs pour une période prolongée, généralement de trois mois ou plus, afin de réduire la fréquence des visites médicales et d'améliorer l'observance thérapeutique [13,14]. Il a été démontré que la dispensation Multi mois réduit les coûts de déplacement, le temps des patients, décongestionne les cliniques VIH, réduit le temps total passé dans les cliniques de 50%, réduit également le nombre de prescriptions sans changement dans le nombre de patients servis, réduisant ainsi la fatigue des ressources humaines [15]. De même, des études ont montré que cette dispensation est idéale pour des personnes très mobiles, personnes qui ont des horaires de travail très rigides comme en Malawi (34% des hommes ayant interrompu leur traitement étaient des hommes mobiles), en zone rurale du Kenya [16,17].

De plus, la DMM n'entraîne pas une suppression virale plus faible chez les PVVIH sous TARV d´après des études au Nigeria (les taux de charge virale supprimée varient de 64% à 92% suite à l´implémentation de la stratégie sous multi-mois chez les enfants et les adolescents) et au Congo [14,18]. En outre, ce modèle de dispensation est bénéfique en cas d´urgence de santé publique: exemple cas de l´épidémie de la COVID-19, étude réalisée en Afrique subsaharienne, en Ouganda, au Nigeria par PEPFAR sur 26 pays [19-22]. Cependant, nous constatons que cette dispensation présente des limites dont nous pouvons citer : risque de non-observance, difficulté à ajuster le traitement, suivi médical insuffisant, risque d´oubli, une complaisance conduisant à un rebond de la charge virale. En plus de compromettre la santé des PVVIH, le rebond viral augmente le risque de transmission du virus et peut conduire à l'échec du traitement et à la résistance aux médicaments [2,23]. Encore En plus de compromettre la santé des PVVIH, le rebond viral augmente le risque de transmission du virus et peut conduire à l'échec du traitement et à la résistance aux médicaments. Des études menées dans d'autres pays ont identifié plusieurs facteurs associés au rebond viral, notamment l'observance thérapeutique, la présence de tuberculose active, la durée de la suppression virale antérieure et des variables socio-économiques et psychosociale [10,24]. Cependant, aucune étude n'a encore évalué ces facteurs chez les patients sous dispensation Multi-mois au Cameroun. Dans cette étude, nous nous sommes proposé d´évaluer le rebond viral et les facteurs associés chez les patients VIH sous traitement antirétroviral à l'Hôpital Régional Annexe de Dschang (HRAD). En analysant ces données, nous espérons fournir des informations précieuses pour améliorer la prise en charge du VIH/SIDA dans la région et contribuer aux efforts mondiaux visant à atteindre les objectifs de l'Onusida pour 2030.

## Méthodes

**Type d´étude:** il s´agissait d´une étude analytique de type cohorte rétrospective sur une durée sur une durée de 08 mois, du de novembre 2023 à juillet 2024.

**Cadre d´étude:** cette étude a été menée au sein de l´Hôpital Régional Annexe de Dschang, situé dans la région de l´Ouest du Cameroun plus précisément dans le district de santé de Dschang. Le personnel de l´HRAD est composé de : médecins généralistes et spécialistes, les infirmiers, les sage-femmes, les aides-soignants, les techniciens de laboratoire, les agents d´entretiens, l´agent d´affaire sociale et le technicien en informatique. Cet hôpital offre une prise en charge médicale complète pour tous les âges et toutes les spécialités, avec des services dédiés à l'accueil des urgences, à l'imagerie médicale, à la santé reproductive, aux maladies ORL, à la médecine générale, à la pédiatrie, à la maternité et à la vaccination, à la réanimation, à la chirurgie, aux nouveau-nés, à la vue, aux troubles mentaux, aux soins dentaires, à la rééducation, à la santé et gynécologique et bien d´autres Dispose également d´unité spécialisée dans le traitement du VIH/SIDA. L´Unité de prise en charge du VIH/SIDA (UPEC) de l´HRA est l´un des centres de référence du district de santé de Dschang. Il présente une grande file active: 1509 PVVIH avec beaucoup de dispensation multi-mois, un bon remplissage des données et pratique des prélèvements de tests de charges virales lors de la prise en charge.

**Population d´étude:** il s´agissait de tous les PVVIH sous traitement à l´Hôpital Régional Annexe de Dschang pendant la période d´étude (janvier 2018 à décembre 2023).

**Critères d´inclusion et d´exclusion:** sont inclus dans l´étude l´ensemble des PVVIH sous dispensation multi-mois et mensuelle, les PVVIH qui ont atteint la suppression virale (CV < 1000 copies/ml) au premier test de charge virale et qui ont 15 ans et plus. Étaient exclus, les PVVIH dont le dossier avait des données manquantes.

**Échantillonnage et taille de l´échantillonnage:** l´échantillonnage était exhaustif. Nous avons recensé auprès de tous les PVVIH sous traitement ARV de la cohorte 2018 à 2023 dans l´HRAD, puis sélectionné les patients en fonction des critères d´inclusion et d´exclusion avant de passer à la collecte de données.

**Calcul de la taille d´échantillon:** nous avons utilisé la formule de Lorentz pour le calcul de la taille minimale de l´échantillon.


$$n=\frac{Z_{\alpha }^{2}P\left(1-p\right)}{d^{2}}$$

Où, n= taille d´échantillon; P=prévalence (p= 19,11%) [11]; d = précision (d= 0,05); Z: fractile de la loi normale pour 95% de confiance (Z=1,96); R= taux de non réponse (R=20%);


$$n=\frac{\left(1,96^{2}\right)\times 0,263\left(1-0,263\right)}{\left(0.05^{2}\right)}$$

Après application numérique, la taille minimale est de 238 participantes. En estimant le taux de dossier mal rempli à 10% on obtient une taille minimale 262 de participantes et en considérant l´effet grappe à 2 degrés on obtient une taille minimale 524 de participants.

## Résultats

**Caractéristiques sociodémographiques et clinique des populations d´études:** au total 519 (438 sous DMM et 81 sous DM) patients âgées de 16ans a 91ans ont été inclus dans notre étude, la tranche d´âge de 30-45 ans était majoritairement représentée dans les deux groupes (42,0% chez les DMM et 48,1% chez les DM valeur p=0,515). De plus les célibataires étaient majoritairement représentés soit 50,57% contre 44,30%, p=0,344 et les patients ayant un niveau d´étude secondaire étaient plus représentés soit 40,2% chez les DMM contre 32,9% chez les DM p-value= 0,253 et majoritairement présent dans la région de l´Ouest soit 88,5% et 94,9% p-value=0,03 respectivement chez les DMM et DM. Aucune différence n'a été observée entre les deux groupes en ce qui concerne le sexe (p= 0,95). Notre étude a montré que 10,1% des DMM contre 14,5% chez les DMM P= 0,426 consommaient de la drogue en outre 53,5% des DMM contre 48,1% des DM consommait de l´alcool p= 0,127.

**Évolution de la couverture de la stratégie multi-mois dans le DS Dschang de 2018 -2023:** sur les 859 PVVIH de la cohorte 2018 à 2019, il apparaît que le taux de couverture de la stratégie multi-mois a fluctué au fil des années; il est passé de 76% en 2018 à 54% en 2020, soit une baisse de 22%. De 2020 à 2022, ce taux a connu une augmentation de 17,6%, passant de 54% à 67,6% avant de baisser de nouveau en 2023, soit 51,02%.

**Incidence du rebond de la charge virale chez les personnes vivant avec le VIH sous dispensation ARV multi-mois et dispensation mensuelle:** il a été découvert que sur les 533 patients, 132 ont eu un rebond de la charge virale, soit 25,43% (132/519). 23,28% (102) des PVVIH sous DMM présentaient un rebond viral contre 37,03% (30/80) chez les PVVIH sous DM (Tableau 1). En outre, OR=1,590 avec IC 95% (1,532-3,257) et p = 0,001, cela signifie que les PVVIH sous DM ont environ 1,59 fois plus de risque de présenter le rebond de la charge virale que les PVVIH sous DMM. L´intervalle de confiance ne contient pas 1 et p-value est inférieure à 0,05. Cela montre une association significative entre le type de dispensation et l´apparition du rebond de la charge virale.

**Tableau 1:** déterminants du rebond de la charge virale chez les PVVIH sous DMM ARV l’HRAD de 2018 à 2023

			Analyse bivariée			Analyse multivariée		
Variable	Rebond de la charge virale		OR	P	IC 95%	ORa	P	IC 95%
	Oui	Non						
**Type de dispensation**								
DMM	102	356	2,55	0,001	1,53-4,25	3,06	0,001	1,74- 5,37
DM	30	51	Ref	Ref	Ref			
**Age du patient (ans)**								
15 - 29	29	88	1,05	0,860	0,61-1,79			
30 - 45	37	186	1,73	0,026	1,06-2,82	1,86	0,014	1,13- 3,06
>45	46	133	Ref	Ref		Ref	Ref	Ref
**Alcool**								
Oui	70	183	2,18	0,001	1,38- 3,44	1,57	0,088	0,93- 2,64
Non	34	194	Ref	Ref		Ref	Ref	Ref
**Tabac**								
Oui	21	31	2,92	0,001	1,59- 5,35	2,48	0,008	1,26- 4,90
Non	80	345	Ref	Ref		Ref	Ref	Ref
**Arrêt du traitement**								
Oui	17	1	58,84	0,001	7,14- 484,93	69,91	0,001	8,19;- 596,14
Non	13	45	Ref	Ref		Ref	Ref	Ref

OR: odds ratio; ORa: odds ratio ajusté; Ref: référence; DM: dispensation mensuelle, DMM: dispensation multi-mois; IC: intervalle de confiance; P: p-valeur

**Facteurs associés du rebond de la charge virale chez les personnes vivant avec le VIH sous dispensation ARV multi mois et Mensuelle:** parmi les caractéristiques sociodémographiques et cliniques chez les PVVIH sous DM, il a été découvert après une régression logistique que le type de dispensation [ORa=3,063, IC à 95%: 1,745-5 ,377,p=0,001], l´arrêt de traitement [ORa=69,90, IC95%: 8,198 - 80, 48 p=0,001], la tranche d´âge de 30-45 ans [ORa=1,862, IC95%: 1,132- 3,062, p=0,001] et la consommation du tabac [ORa=2,488, IC95%: 1,263 -4,903, p=0,008] étaient significativement associés à un rebond viral après régression multivariée (Tableau 1).

## Discussion

**L´évolution de la couverture de la stratégie multi-mois:** parmi les 859 patients inclus dans l'étude, une tendance instable a été observée dans l'évolution du taux de couverture de dispensation des antirétroviraux au fil des années. En 2018, le taux était de 76%, mais il a légèrement diminué en 2019 pour atteindre 74,30%. Cette baisse pourrait être attribuée à des problèmes logistiques ou à des pénuries de médicaments. En 2020, le taux a chuté de manière significative à 54%, ce qui représente une baisse considérable par rapport aux années précédentes. Cette baisse pourrait être liée à la pandémie de COVID-19, qui a affecté la disponibilité des médicaments et l'accès aux soins de santé, expliquant en partie cette diminution. L'OMS a signalé que vingt-quatre pays ont rencontré des difficultés d'approvisionnement en ARV pendant la pandémie de COVID-19, en raison de retards de livraison des médicaments et de perturbations des services de transport terrestre et aérien, ainsi que d'un accès restreint aux services de santé à l'intérieur des pays [25].

**Incidences du rebond de la charge virale:** parmi les 533 patients inclus dans notre étude, 132 ont présenté un rebond de la charge virale, ce qui correspond à un taux d'incidence global de 25,6%. Les taux spécifiques de rebond étaient de 23,28% chez les patients sous dispensation multi-mois (DMM) et de 37,03% chez ceux sous dispensation mensuelle (DM). Les patients DMM et DM peuvent présenter des caractéristiques démographiques différentes, ce qui pourrait expliquer la différence observée. Dans notre cas, 48,1% des patients sous DM sont de la tranche de 30-45 ans qui est la tranche associée au rebond, contre 42% chez les DMM. Notre incidence est identique à l'étude menée en 2008 par Geretti *et al*. a un taux de rebond 25,5% [26]. Notre incidence virologique est plus élevée que celle rapportée dans deux études menées: au Ghana par opoku *et al*. en 2022, qui a trouvé une incidence de 21% et au Cameroun par Cavin Bekolo *et al*. en 2024, qui avait pour incidence 19,11 pour 1000 [10,11]. Mais inférieure à celles observées dans des études menées au Royaume-Uni par Harrigan *et al*. en 1999, au Kenya par Maina *et al*. en 2020, Mocroft *et al*. en 2003 et avec des incidences de 28,06%, 49%, 42,2% et 32% respectivement [2,1,2,27]. Ces variations peuvent être expliquées par les différences démographiques et au niveau de la taille d´échantillon entre les populations étudiées, comme dans notre cas où nous avons plusieurs tailles d´échantillon et des études qui se sont faites.

### Facteurs associes au rebond de la charge virale

**Type de dispensation:** la dispensation multi-mois peut poser des défis pour les patients ayant un accès limité aux soins de santé, notamment en termes de stockage des médicaments (un stockage inapproprié peut affecter l'efficacité des ARV) et augmenter le risque d'oubli de prise des médicaments étude de Msosa *et al*. en 2024, [28]. De plus, les patients sous dispensation multi-mois pourraient être moins attentifs aux effets secondaires potentiels du traitement, car ils prennent les médicaments sur une période plus longue. La non-détection de ces effets secondaires pourrait les inciter à arrêter le traitement, ce qui pourrait conduire à un rebond viral [29].

**Âge:** les patients âgés de 30 à 45 ans présentant un risque accru (p=0,014). Cette observation est cohérente avec les résultats d'une étude menée au Kenya par Maina *et al*. en 2020, qui a montré une association entre la tranche d'âge de 40-59 ans et le rebond de la charge virale (p=0,002) [2]. Les différences d'âge observées dans les associations avec le risque de rebond viral pourraient être attribuées à des variations dans les populations étudiées et les méthodologies employées. En effet, notre étude et celle menée au Kenya présentent des tailles d'échantillons différentes, et l'étude au Kenya a été réalisée dans plusieurs centres hospitaliers (Malindi et Meru). La tranche d'âge 30-45 ans pourrait être davantage associée au rebond viral en raison des pressions professionnelles et familiales qui peuvent affecter l'observance thérapeutique. Il est crucial de prendre en compte les facteurs socio-économiques et environnementaux qui peuvent influencer la capacité des patients à maintenir une adhérence optimale au traitement, comme l'ont souligné Jin *et al*., en 2008 et Martin en 2022 [30,31].

**Consommation de tabac:** cette observation est en ligne avec les travaux de Julia H en 2002 qui ont démontré qu'une faible proportion (13%) des PVVIH utilisant des drogues parvenaient à maintenir une charge virale indétectable, les autres connaissant des épisodes de rebond [24]. La consommation de tabac pourrait aggraver la situation en affaiblissant le système immunitaire des PVVIH, les rendant plus vulnérables aux effets du VIH et diminuant potentiellement l'efficacité des traitements antirétroviraux. Des études menées à Bamako en 2013 (Dao *et al*.), aux États-Unis en 2022 (Bhalerao *et al*.) et au Cameroun en 2022 (Esther Voundi *et al*.) ont confirmé ces observations [32-34].

**Arrêt du traitement:** nous avons observé une forte association p=0,001 entre l´arrêt du traitement et le rebond virologique. Cette association est cohérente avec plusieurs autres recherches menées à Paris par Palich *et al*., en 2017 qui nous présente un rebond de charge virale dans le sperme après un arrêt de traitement ainsi qu´aux États unis par Garcías F *et al*. en 1999 qui nous montre un rebond de la charge virale chez huit patients. Une étude par Macroft *et al*. en 2003 dans les pays de Europe SIDA montre que les patients ayant arrêté le traitement ont connu un rebond soit un taux de 42,2%, Neumann *et al*. en 1999 et en fin l´étude Harigina *et al*. où après arrêt du traitement on observe un rebond chez tous les 6 homosexuels [13,27,35-37]. Cent pour cent (100%) des patients qui arrêtent leur traitement présentent un rebond viral, mais le délai d'apparition varie selon les individus et leurs caractéristiques. Il est fort probable que l´arrêt du traitement soit un facteur significatif dans le rebond de la charge virale cela pourrait être dû à plusieurs raisons comme: les effets secondaires mal tolérés, des difficultés d´accès au médicament, peut également favoriser l´émergence de souches résistantes au virus [38].

**Limites de l´étude:** cette étude est réalisée dans un hôpital de référence. On ne saurait généraliser les résultats. Par ailleurs, les données manquantes dans les dossiers (biais d´information) et les données de revue insuffisantes sur la dispensation multi mois ont été des limites à cette étude. Des données complémentaires sur la dispensation multi-mois aurait pu nous donner de meilleures précisions étiologiques. Néanmoins, ces résultats sont le reflet du rebond virologique de la population du District de Santé de Dschang.

## Conclusion

Notre étude a eu pour but d´étudier le rebond virologique et les facteurs associés chez les PVVIH sous traitement ARV suivis à l´HRAD. L´ensemble de nos patients ont été sous traitement antirétroviral où on observe une diminution significative du taux de couverture de la stratégie multi-mois entre 2018 et 2023. Les 519 patients inclus dans notre étude, 132 n´arrivaient pas à maintenir les charges virales supprimée à long terme, l´incidence de rebond virale global est de 21,8. Il en ressort que ces PVVIH n´arrivent pas à annuler leurs charges virales à long terme en raison du style de vie (consommation d´alcool, consommation de tabac), des problèmes d´observance thérapeutique, des caractéristique sociodémographiques. Nous recommandons au Ministère de la Santé publique de promouvoir l'adhésion à la DMM pour maximiser ses bénéfices et d'améliorer l'observance chez les personnes âgées de 30 à 45 ans. Le personnel de santé doit sensibiliser les PVVIH sous DMM à mieux suivre leur traitement et à éviter les facteurs de risque de rebond virologique, tels que le tabagisme et l'interruption du traitement. Les PVVIH doivent suivre les recommandations médicales, tandis que les chercheurs sont encouragés à explorer l'impact de la stigmatisation sociale sur l'adhésion au traitement.

### Etat des connaissances sur le sujet

- La dispensation multi-mois n'entraîne pas une suppression virale plus faible chez les personnes vivant avec le VIH sous TARV;
- Les taux de rebond viral varient considérablement d'une région à l'autre, avec des incidences rapportées de 21% au Ghana, 40% au Kenya, 28,06% au Royaume-Uni et 19,11 pour 1000 au Cameroun;
- Des études révèlent que plusieurs facteurs sont associés au rebond viral, notamment l'observance thérapeutique, la présence de tuberculose active, la durée de la suppression virale antérieure et des variables socio-économiques et psychosociale.

### Contribution de notre étude à la connaissance

- Le taux de couverture de la stratégie multi-mois était de 51,02% en 2023 à l’HRAD;
- L’incidence de rebond viral global était de 21,8 %, avec 18,8% chez les dispensation multi-mois et 37,8% chez les DM;
- La tranche d’âge de 30-45 ans, le type de dispensation (DMM), la consommation du tabac et d’alcool sont les facteurs qui ont conduit à des épisodes de rebond chez les personnes vivant avec le VIH sous TARV.
